# Development of nucleic acid-based RAA test strip assay for the rapid detection of foot-and-mouth disease virus

**DOI:** 10.3389/fvets.2025.1526005

**Published:** 2025-06-23

**Authors:** Wenxian Chen, Xinni Niu, Weijun Zeng, Yiqi Fang, Zixiang Zhu, Lin Yi, Mingqiu Zhao, Hongxing Ding, Shuangqi Fan, Zhaoyao Li, Jinding Chen

**Affiliations:** ^1^College of Veterinary Medicine, South China Agricultural University, Guangzhou, China; ^2^State Key Laboratory for Animal Disease Control and Prevention, College of Veterinary Medicine, Lanzhou Veterinary Research Institute, Lanzhou University, Chinese Academy of Agricultural Sciences, Lanzhou, China; ^3^Wen’s Foodstuffs Group Co., Ltd., Yunfu, China

**Keywords:** foot-and-mouth disease virus, isothermal amplification, recombinase-aided amplification, nucleic acid test strip, rapid detection

## Abstract

Foot-and-mouth disease virus (FMDV) causes blister-like lesions in animals, leading to significant economic losses in animal husbandry. Accurate and rapid detection of FMD is crucial for effective prevention and control. The recombinase-aided amplification (RAA) technique enables rapid amplification of target fragments under isothermal conditions. In this study, based on the conserved sequence of the FMDV 3D gene, optimal RAA primers and probes were designed and screened, and a simple, rapid, and visual FMDV nucleic acid RAA test strip was developed. The optimum reaction conditions of the assay were determined to be 32°C for 30 min. The specificity, sensitivity, and repeatability of the FMDV nucleic acid RAA test strip were evaluated. The results demonstrated that the FMDV nucleic acid RAA test strip specifically reacted with FMDV nucleic acid and exhibited no cross-reactivity with other viruses. The lowest detection limits for recombinant plasmids and virus titer were 10 copies/μL and 10^0^ TCID_50_/mL, respectively. In addition, all 17 positive samples and 21 negative samples were accurately identified using the FMDV nucleic acid RAA test strip, resulting in a 100% positive detection rate. In conclusion, the FMDV nucleic acid RAA test strip developed in this study—characterized by high specificity, efficiency, and sensitivity—offers a robust technical platform for the prevention and control of FMD.

## 1 Introduction

Foot-and-mouth disease (FMD), a highly contagious disease caused by the FMDV, poses a significant threat to cloven-hoofed animals, including domestic and wild ruminants, resulting in substantial economic losses in animal husbandry (1–3). Owing to its high genetic variability, FMDV has evolved into seven distinct serotypes (O A, C, Asia 1, SAT 1, SAT 2, and SAT 3), with over 100 serosubtypes (4, 5). At present, FMDV serotype O is one of the predominant prevalent strains in our country (6). Animals often develop fever, claudication, excessive salivation, and characteristic blistering on the hooves and in the mouth after FMDV infection. In young animals, FMDV infection can lead to sudden death due to acute myocarditis (7, 8). Moreover, FMDV can contaminate the surroundings through aerosols and cause long-distance transmission events, which complicates the control of FMD outbreaks (9). Although prevention and control measures—such as vaccine vaccination, daily testing, strict quarantine, and the culling of infected animals—have yielded some success in reducing FMD outbreak, we still need to be vigilant for sporadic outbreaks and the risk of FMDV strain from abroad. In addition, clinical diagnosis alone cannot reliably differentiate FMDV from other viruses that cause similar vesicular symptoms, such as Swine vesicular disease virus (SVDV) and Senecavirus A (SVA) (10). Therefore, it is necessary to develop a fast and sensitive FMDV diagnostic method to provide efficient technical means for its prevention and control.

Recombinase-aided amplification (RAA) is a novel isothermal amplification technique based on recombinase polymerase amplification (RPA) technology, which has been widely used in the detection of a variety of pathogens. The principle of RAA is similar to that of RPA, but the recombinant enzyme in RAA is derived from *E. coli* rather than T4 phage (11, 12). The sequence length of RAA primers should be 30–35 bp, and the length of target fragments should be controlled within 100–500 bp. During the RAA reaction, a recombinant enzyme binds to the primer and anchors to the cDNA template chain. With the help of single-stranded DNA-binding protein (SSB) and DNA polymerase, the template chains are progressively unraveled, and new nucleic acid chains are continuously synthesized. RAA reaction enables rapid nucleic acid amplification within 30 min under isothermal conditions (37–42°C), eliminating the need for specialized thermal cycling equipment (13). Moreover, the product labeled with 6-carboxy-fluorescein (FAM) and biotin can be generated through the RAA reaction using an added probe. This product binds to specific antibodies on the disposable nucleic acid test strip, enabling rapid visual readout of results. Specifically, the test strip contains three functional zones (13): (1) a conjugate pad pre-loaded with colloidal gold particles conjugated to anti-FAM antibodies, (2) a nitrocellulose membrane containing a test line (T line) coated with streptavidin and a control line (C line) immobilized with secondary anti-FAM antibodies, and (3) an absorbent pad. When the dual-labeled RAA amplicons are applied to the sample pad, the FAM moiety binds to the anti-FAM-gold nanoparticle complexes, forming a mobile immunocomplex. This complex migrates laterally along the test strip via capillary action toward the nitrocellulose membrane. Upon reaching the T line, the biotin tag on the amplicon is captured by streptavidin, resulting in the accumulation of gold nanoparticles and the formation of a visible red band. Excess complexes continue to flow to the C line, where the anti-FAM antibodies bind the gold conjugates, confirming proper strip function. A positive result is indicated by red bands appearing at both the T and C lines within 10–15 min, while only the C line appears in negative samples. At present, RAA nucleic acid test strip detection technology has been applied in the field diagnosis of various pathogens due to its simple primer design, rapid amplification kinetics, and high sensitivity. Wang et al. successfully developed HCV RAA-side flow test strips for hepatitis C virus (HCV) detection, which could complete the reaction within 30 min with a minimum plasmid detection limit of 10 copies/μL (14). An RT-RAA-LFD detection method based on the HA gene of Avian influenza viruses (AIV) was also constructed, and the method could complete the reaction within 30 min at 39°C (15), with a minimum detection limit of 10 copies/μL and sensitivity comparable to that of RT-qPCR. These findings indicate that combining RAA with nucleic acid test strips constitutes a rapid, visual detection system with high sensitivity and strong specificity, showing great potential for the clinical diagnosis of FMDV. To address the limitations of conventional diagnostic methods, this study aimed to develop a rapid, sensitive, and field-deployable FMDV nucleic acid RAA test strip for on-site detection, eliminating reliance on centralized laboratory infrastructure.

## 2 Materials and methods

### 2.1 Virus and plasmid

The FMDV serotype O strain O/BY/CHA/2010 was propagated in Baby Hamster Kidney-21 (BHK-21) cells using standard virology techniques, and the supernatants of infected cells were clarified and stored at −80°C. The genomic DNA or cDNA of SVA, SVDV, porcine parvovirus (PPV), porcine circovirus type 2 (PCV2), classical swine fever virus (CSFV), pseudorabies virus (PRV), and porcine reproductive and respiratory syndrome virus (PRRSV) was deposited by the Department of Microbiology and Immunology, College of Veterinary Medicine, South China Agricultural University, Guangzhou, China. FMDV 3D gene (GenBank: JN654459) was synthesized and cloned into the pMD19-T vector. The recombinant plasmid pMD19-T-FMDV was extracted using E.Z.N.A Plasmid Mini kit I (OMEGA, USA).

### 2.2 RCR and qPCR for FMDV detection

Conventional PCR detection primers for the FMDV 3D gene, with an amplified fragment length of 516 bp, were used as previously described (16). A total of 22 μL of Golden Star T6 Super PCR Mix (Beijing Tsingke Biotechnology Co., Ltd., China), 1 μL of each primer (10 μM), and 1 μL of cDNA were mixed evenly, and the mixture was subjected to PCR. The PCR amplification was performed under the following conditions: initial denaturation at 98°C for 2 min, followed by 30 cycles of 98°C for 15 s, 57°C for 15 s, 72°C for 30 s, with a final extension at 72°C for 2 min. The amplicons were analyzed using 1% agarose gel electrophoresis.

Specific primers for qPCR detection of the FMDV 3D gene were also used previously described (16), with the amplified target gene length of 200 bp. The qPCR reaction mixture consisted of 20 μL total volume, including 10 μL of ChamQ SYBR qPCR Mix (Vazyme Biotech Co., Ltd., China), 0.4 μL of each primer (10 μM), 8.2 μL of ultrapure water, and 1 μL of cDNA. The qPCR amplification was performed under the following conditions: pre-denaturation at 95°C for 30 s, followed by 35 cycles of denaturation at 95°C for 10 s, and annealing at 60°C for 30 s.

### 2.3 Design of RAA primers and probe

According to the principle of primer design for the RAA nucleic acid amplification kit (Jiangsu Qitian Gene Biotechnology Company Limited, China), Primer Premier 5.0 and Oligo 7 software were used to design multiple RAA primer pairs for the FMDV 3D gene conserved region. The designed primers were screened via RAA agarose gel electrophoresis using FMDV cDNA as the template and ultrapure water as a negative control. The reaction system and procedure were as follows: 25 μL of buffer V, 15 μL of ultrapure water, 2 μL of forward primer (10 μM), 2 μL of reverse primer (10 μM), and 1 μL of cDNA sample were added to the reaction tube containing lyophilized enzyme powder and mixed thoroughly. A total of 5 μL of magnesium acetate solution (280 mM) was then added to the reaction tube. The tube was incubated at 37°C for 30 min. After amplification, the product was mixed completely with an equal volume of chloroform until a white precipitate appeared. The mixture was then centrifuged at 12,000 rpm for 5 min at room temperature, and the supernatant was collected for analysis using 1.5% agarose gel electrophoresis.

After selecting the optimal primer pair based on RAA agarose gel electrophoresis analysis, an RAA probe was designed according to the principle of RAA probe design by Beacon Designer 7 software, and specific biomarkers were added to the primers and probes. Specifically, the RAA probes were 46–50 bp in length, with FAM labeled at the 5′ end, a tetrahydrofuran residue site (THF) 30 nucleotides downstream of the 5′ end, and a block group (C3 spacer) at the 3′ end. Biotin labeling was added to the 5′ end of the primer complementary to the probe sequence. All primers and probes were synthesized by Sangon Biotech (China).

### 2.4 Establishment of the FMDV nucleic acid RAA test strip

After obtaining the best RAA primers and probes, the nucleic acid RAA test strip method was established for FMDV detection using FMDV cDNA as a template and ultrapure water as a negative control. The reaction system and procedure were as follows: a mixture containing 25 μL of buffer V, 13.4 μL of ultrapure water, 2 μL of forward primer (10 μM), 2 μL of reverse primer (10 μM), 0.6 μL of probe (10 μM), and 2 μL of DNA sample was added to the reaction tube containing lyophilized enzyme powder and mixed evenly. A total of 5 μL of magnesium acetate solution (280 mM) was then added to the reaction tube. The reaction tube was incubated at 37°C for 30 min. After amplification, 10 μL of RAA product was dropped into the sample pad of the nucleic acid test strip, and the strip was then incubated in tubes containing 100 μL of ultrapure water for 5–10 min. Both the quality control line (C) and the test line (T) observed on the nucleic acid test strip were judged as positive, whereas the negative result only displayed a C line. The results were invalid if the C line was not shown in colors.

### 2.5 Optimization of reaction condition for the FMDV nucleic acid RAA test strip

To obtain the optimal reaction temperature for the FMDV nucleic acid RAA test strip, the RAA reaction system was incubated in a water bath at different temperatures (25–45°C) for 30 min. The optimum temperature was determined based on the color rendering degree of the T line of the test strip. Subsequently, the RAA reaction system was incubated in a water bath at the optimal temperature for varying times (5–35 min) to verify the optimal incubation time of the FMDV nucleic acid RAA test strip.

### 2.6 Specificity, sensitivity, and repeatability of the FMDV nucleic acid RAA test strip

The specificity of the FMDV nucleic acid RAA test strip was evaluated using various swine-associated viruses, including SVA, SVDV, PPV, PCV2, CSFV, PRV, and PRRSV. The sensitivity of the FMDV nucleic acid RAA test strip method was determined using recombinant plasmid pMD19-T-FMDV (10^0^–10^10^ copies/μL, prepared by 10-fold serial dilution) and FMDV virus solution (10^−2^ to 10^6^ TCID_50_/mL, prepared by 10-fold serial dilution), and then compared with PCR, qPCR, and RAA agarose gel electrophoresis. The minimum concentration gradient of the plasmid was tested repeatedly on the FMDV nucleic acid RAA test strip, and after three replicates, the color rendering results of the nucleic acid test strip were compared to analyze the repeatability of the method.

### 2.7 Preliminary clinical validation of the FMDV nucleic acid RAA test strip

Nucleic acid was extracted from 3 FMDV-inactivated antigens, 14 FMDV-inactivated vaccines, and 21 healthy pig blood samples. The 17 positive samples and 21 negative samples were detected using the FMDV nucleic acid RAA test strip, conventional PCR, and qPCR, respectively.

## 3 Results

### 3.1 Establishment of the FMDV nucleic acid RAA test strip and optimization of the reaction condition

**Figure 1:**
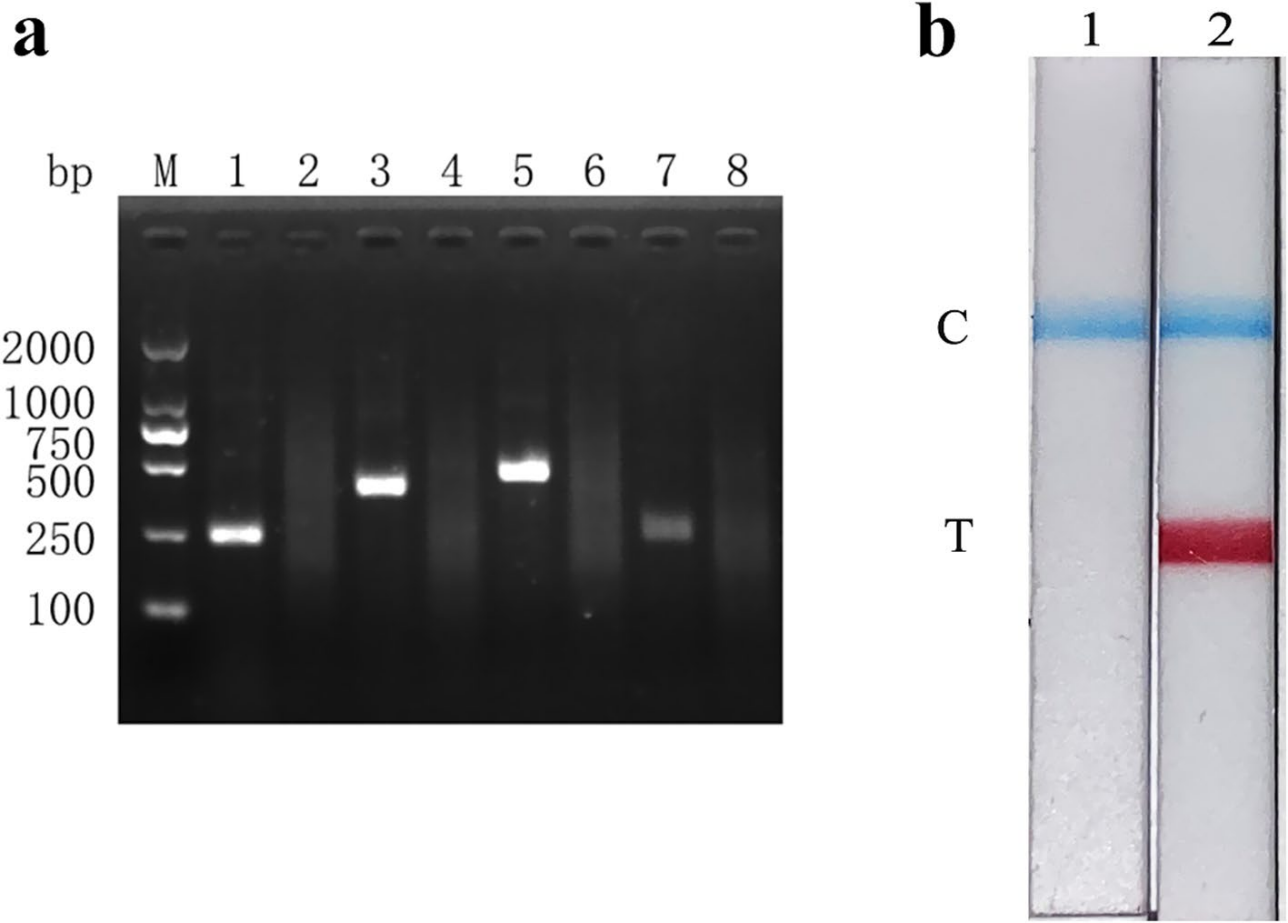
Establishment and initial application of the FMDV nucleic acid RAA test strip method. **(a)** The products amplified by different RAA primers were analyzed using the 1.5% agarose gel electrophoresis to determine the optimal primer pairs. M is a 2,000 bp DNA marker. The primers and templates used in lanes 1–8 successively are: FMDV-RAA-F1/R1 + FMDV, FMDV-RAA-F1/R1 + ddH_2_O, FMDV-RAA-F2/R2 + FMDV, FMDV-RAA-F2/R2 + ddH_2_O, FMDV-RAA-F3/R3 + FMDV, FMDV-RAA-F3/R3 + ddH_2_O, FMDV-RAA-F4/R4 + FMDV, and FMDV-RAA-F4/R4 + ddH_2_O. **(b)** Preliminary development of the FMDV nucleic acid RAA test strip method. The templates used in lanes 1 and 2 are negative control and FMDV cDNA, respectively.

Following the RAA primer design principles, four pairs of specific primers targeting the FMDV 3D gene for RAA amplification were designed and synthesized (Table 1). The designed primers were screened by RAA agarose gel electrophoresis to detect primer specificity. As shown in Figure 1a, all four pairs of FMDV RAA primers produced specific bands at approximately 249 bp, 375 bp, 436 bp, and 250 bp, respectively, which were consistent with the expected sizes. Among these bands, the FMDV-RAA-F2/R2 and FMDV-RAA-F3/R3 primer pairs produced the brightest specific bands. In accordance with the principles of RAA primer design, which prioritize shorter amplicon lengths, the FMDV-RAA-F2/R2 primer pair (375 bp) was selected as optimal for the FMDV RAA assay. The probe was then designed based on the targeted amplification fragment. Details regarding the probe and primers are listed in Table 2.

**Table 1:** FMDV RAA primers used in this study.

Primer name	Sequence (5′-3′)	Length (bp)
FMDV-RAA-F1	TTCTGAAGACCCTCGTGAACACGGAACACGCC	257
FMDV-RAA-R1	TCATAATCACTAGCAACCACGATGTCGTCTCCG	
FMDV-RAA-F2	CTCAAACAACGGACCGCAAATTGGATCAGCGG	375
FMDV-RAA-R2	TCCAGCTCAACTCCCTCATAGTGTCTACGCAGCG	
FMDV-RAA-F3	CTCAAACAACGGACCGCAAATTGGATCAGCGG	436
FMDV-RAA-R3	GTCCAAGTCATAATCACTAGCAACCACGATGTCG	
FMDV-RAA-F4	TTTCCACCCGAATGCTGAGTGGATTCTGAAGACCC	250
FMDV-RAA-R4	GTCCAAGTCATAATCACTAGCAACCACGATGTCG	

**Table 2:** Optimal primer pairs and probes for the FMDV nucleic acid RAA test strip.

Primer name	Sequence (5′-3′)	Length (bp)
FMDV-RAA-B-F3	Biotin-CTCAAACAACGGACCGCAAATTGGATCAGCGG	375
FMDV-RAA-R3	TCCAGCTCAACTCCCTCATAGTGTCTACGCAGCG	
FMDV-P	FAM-CACGTAGATGTTGTTCAGAATTGTGTTGATG- THF-ATGCTGGTTGCGGAA-C3 spacer	

The FMDV nucleic acid RAA test strip method was performed using FMDV cDNA as a template and ultrapure water as a negative control. The results showed that both the C line and the T line appeared colored on the strip with FMDV cDNA, while only the C line appeared (as a blue band) in the negative control (Figure 1b). These results indicate that the FMDV nucleic acid RAA test strip method was preliminarily established.

**Figure 2:**
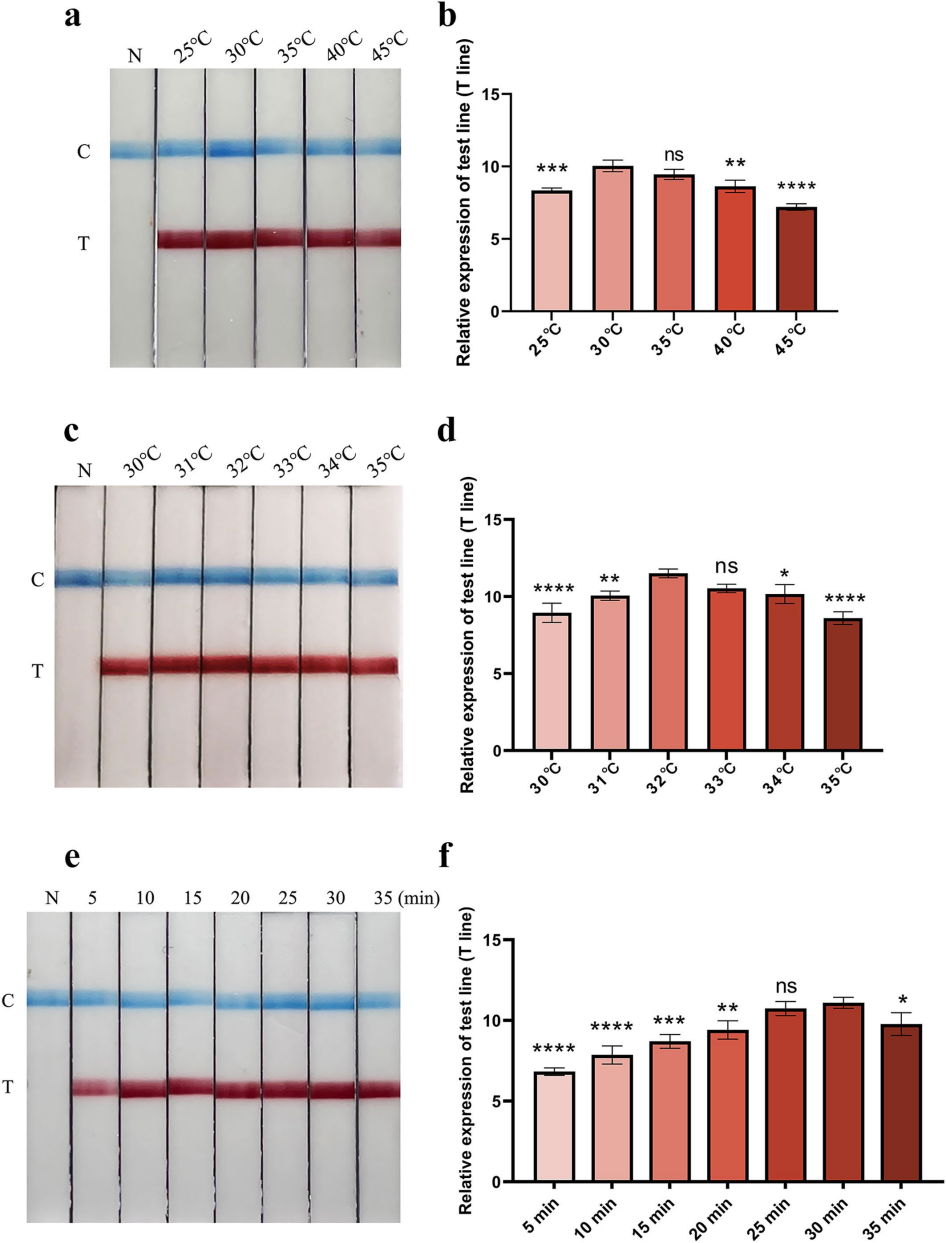
Optimization of reaction temperature and time. **(a,b)** The optimal reaction temperature range of the FMDV nucleic acid RAA test strip. The relative expression of test line (T line) was analyzed using the ImageJ plugin and plotted in GraphPad Prism 9 (right panel). Error bars indicate the mean (± SD) of three independent experiments. ns, *p* ≥ 0.05; *, *p* < 0. 1; **, *p* < 0.01; ***, *p* < 0.001; ****, *p* < 0.0001 (one-way ANOVA). **(c,d)** The accurate optimal temperature detection of the FMDV nucleic acid RAA test strip. The relative expression of T line was analyzed using the ImageJ plugin and plotted in GraphPad Prism 9 (right panel). Error bars indicate the mean (± SD) of three independent experiments. ns, *p* ≥ 0.05; *, *p* < 0. 1; **, *p* < 0.01; ***, *p* < 0.001; and ****, *p* < 0.0001 (one-way ANOVA). **(e,f)** Optimization of reaction time. Relative expression of the T line was analyzed using the ImageJ plugin and plotted in GraphPad Prism 9 (right panel). The error bars indicate the mean (± SD) of three independent experiments. ns, *p* ≥ 0.05; *, *p* < 0. 1; **, *p* < 0.01; ***, *p* < 0.001; ****, *p* < 0.0001 (one-way ANOVA).

To determine the optimum reaction temperature range of the FMDV nucleic acid RAA test strip method, the RAA reaction was incubated at different temperatures (25, 30, 35, 40, and 45°C) for 30 min. The results showed that the suitable reaction temperature range was 30–35°C (Figures 2a,b). The optimum reaction temperature of the RAA assay was further screened, and 32°C was identified as the optimal reaction temperature of the method (Figures 2c,d). Subsequently, the optimal incubation time was evaluated at 32°C. As shown in Figures 2e,f, the T line appeared clearly after the RAA assay was incubated for 30 min. Therefore, 30 min was selected as the optimal incubation time for the FMDV nucleic acid RAA test strip method.

### 3.2 Specificity, sensitivity, and repeatability of the FMDV nucleic acid RAA test strip

**Figure 3:**
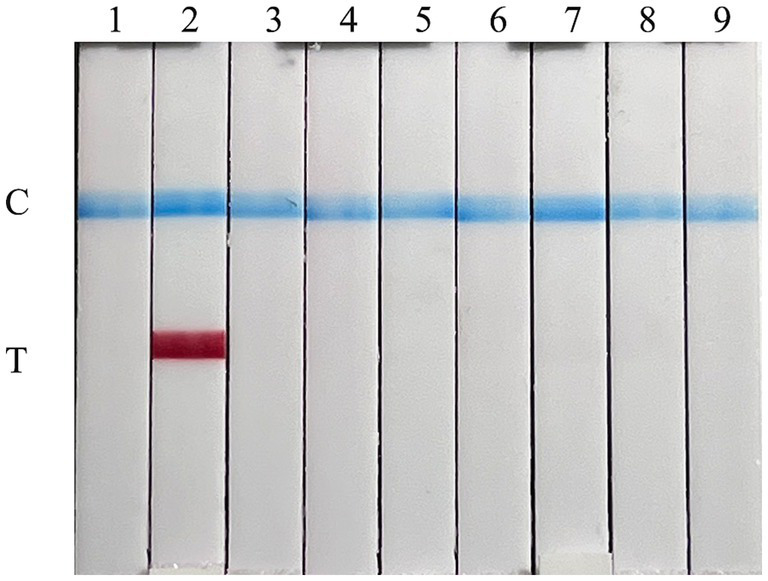
Specificity of the FMDV nucleic acid RAA test strip. The specificity test results of the FMDV nucleic acid RAA test strip using eight different pig viruses. The samples used in lanes 1–9 successively are as follows: ddH_2_O, FMDV, SVA, SVDV, PPV, PCV2, CSFV, PRV, and PRRSV.

The specificity of the FMDV nucleic acid RAA test strip was confirmed using nucleic acids from various viruses as reaction templates. As shown in Figure 3, the T line appeared on the test strip containing FMDV cDNA, while no cross-reaction was observed with other pathogens, indicating that the FMDV nucleic acid RAA test strip is highly specific.

**Figure 4:**
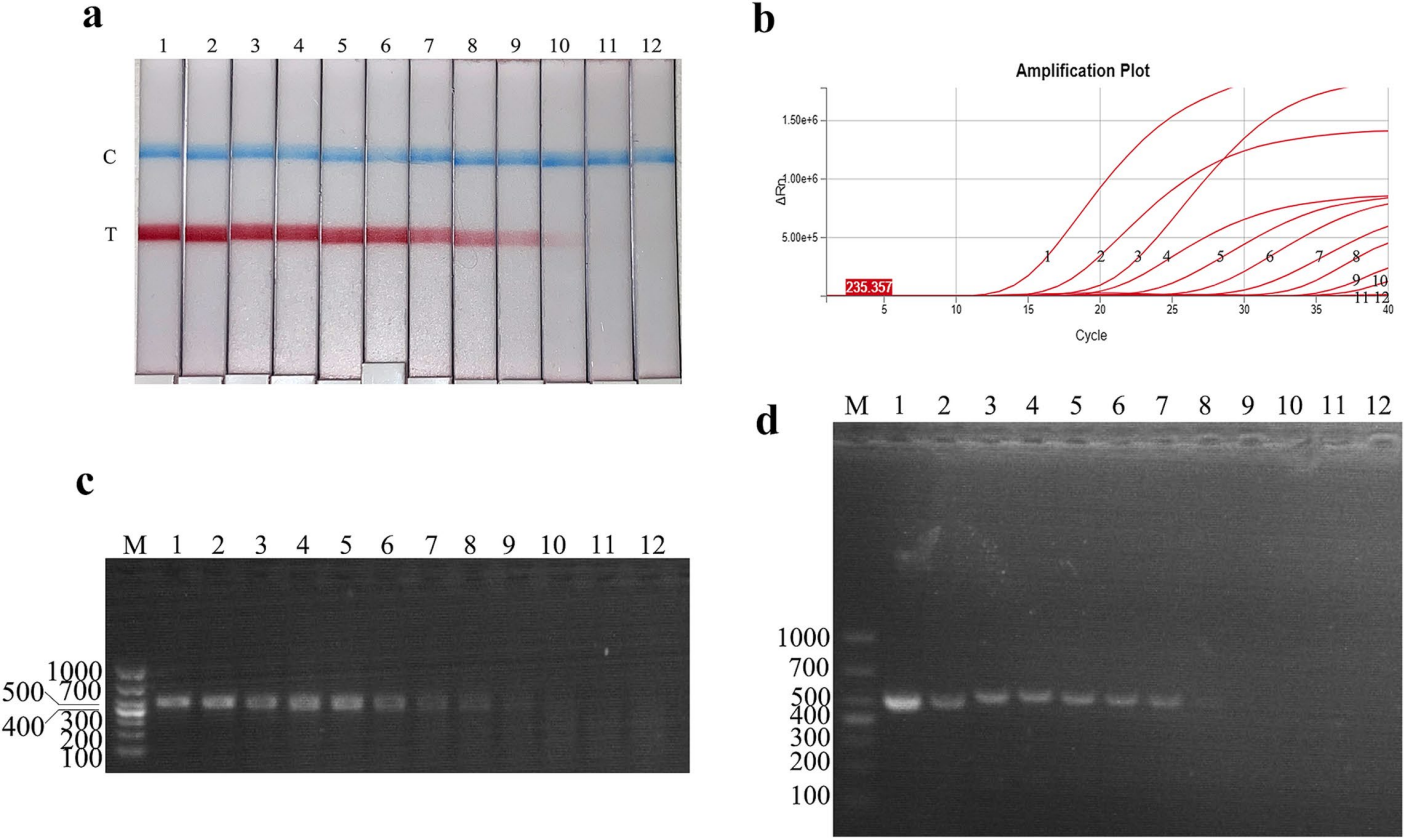
Recombinant plasmid sensitivity analysis of the FMDV nucleic acid RAA test strip. **(a–d)** The sensitivity test results of plasmid copy number using the FMDV nucleic acid RAA test strip, qPCR assay, the RAA agarose gel electrophoresis assay, and the conventional PCR assay. M is a 2,000 bp DNA marker. The copies of templates used in lanes 1–11 successively are 10^10^, 10^9^, 10^8^, 10^7^, 10^6^, 10^5^, 10^4^, 10^3^, 10^2^, 10^1^, and 10^0^ copies/μL. Lane 12 is a negative control.

The plasmid sensitivity of the FMDV nucleic acid RAA test strip was evaluated and compared with PCR, qPCR, and RAA agarose gel electrophoresis. The results showed that the FMDV nucleic acid RAA test strip had a minimum detection limit of 10 copies/μL, matching the sensitivity of the qPCR method and demonstrating 100-fold higher sensitivity than conventional PCR and RAA agarose gel electrophoresis (Figures 4a–d).

**Figure 5:**
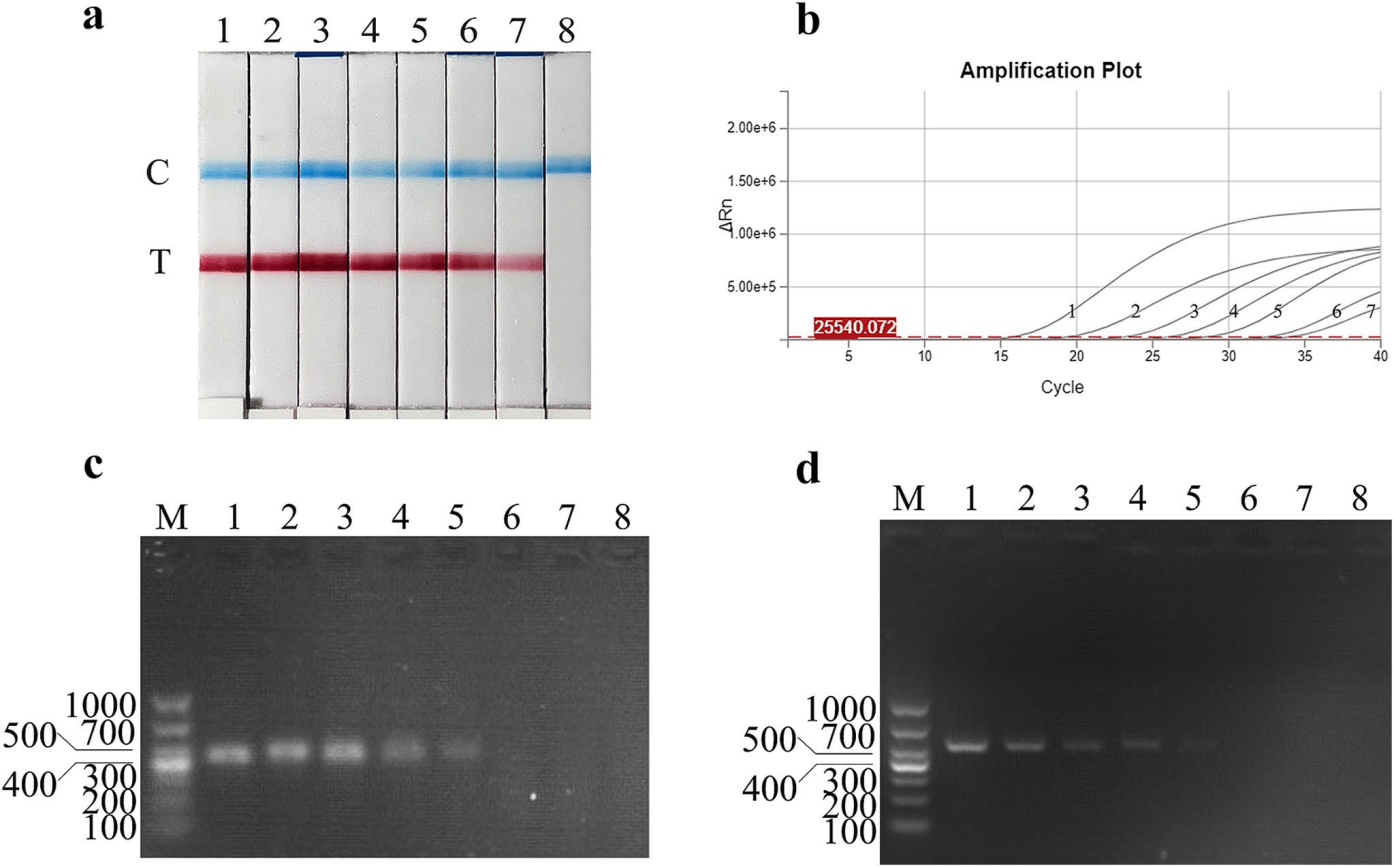
Virus titer sensitivity analysis of the FMDV nucleic acid RAA test strip. **(a–d)** The sensitivity test results of the virus titer using the FMDV nucleic acid RAA test strip, qPCR assay, the RAA agarose gel electrophoresis assay, and the conventional PCR assay. M is a 2,000 bp DNA marker. The TCID_50_ templates used in lanes 1–8 successively are as follows 10^6^, 10^5^, 10^4^, 10^3^, 10^2^, 10^1^, 10^0^, and 10^−1^ TCID_50_/mL.

The virus titer sensitivity of the FMDV nucleic acid RAA test strip was further tested, and the results showed that the FMDV virus titer detection limits for FMDV nucleic acid RAA test strip, qPCR, conventional PCR, and RAA agarose gel electrophoresis were 10^0^ TCID_50_/mL, 10^−1^ TCID_50_/mL, 10^2^ TCID_50_/mL, and 10^2^ TCID_50_/mL, respectively (Figures 5a–d).

**Figure 6:**
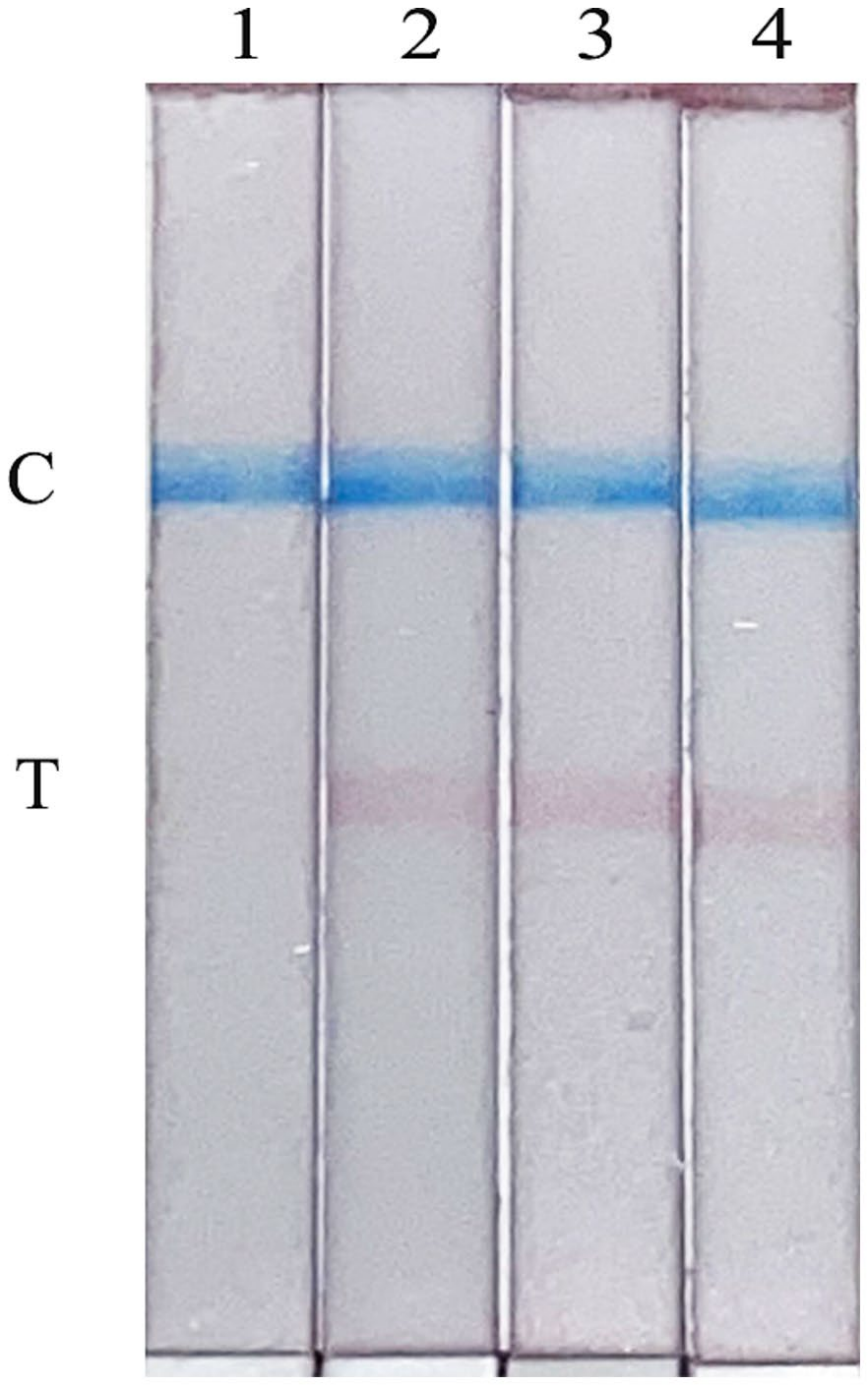
Repeatability of the FMDV nucleic acid RAA test strip. Lane 1 is a negative control. The copies of templates used in lanes 2–4 are 10 copies/μL.

To evaluate the stability of the FMDV nucleic acid RAA test strip established in this study, the reproducibility test was performed on a standard plasmid with a concentration of 10 copies/μL. As shown in Figure 6, the FMDV nucleic acid RAA test strip has good stability and repeatability.

### 3.3 Clinical sample detection

The performance of the FMDV nucleic acid RAA test strip was evaluated using 17 positive samples and 21 negative samples. The results showed that (Table 3) 17 positive samples and 21 negative samples were correctly detected upon utilizing the FMDV nucleic acid RAA test strip and qPCR method. The two methods have a 100% positive coincidence rate. A total of 15 positive samples and 23 negative samples were detected using conventional PCR and RAA agarose gel electrophoresis. The positive coincidence rate of these two methods was 88.24%, and the negative coincidence rate was 91.3%. It shows that the FMDV nucleic acid RAA test strip method has certain clinical feasibility.

**Table 3:** Comparison of sample detection results by different detection methods.

Detection method	Positive samples	Negative samples
Conventional PCR	15	23
RAA agarose gel electrophoresis	15	23
qPCR	17	21
FMDV nucleic acid RAA test strip	17	21

## 4 Discussion

FMD is a highly contagious viral disease that poses significant threats to cloven-hoofed animals. Current control strategies rely on surveillance programs, culling of infected herds, and mass vaccination campaigns (17). However, FMDV-induced vesicular lesions are clinically indistinguishable from those caused by other vesiculoviruses, such as SVA and SVDV, necessitating laboratory confirmation for accurate diagnosis (18–20). At present, the main molecular biological methods for FMDV detection include PCR and qPCR, which require expensive thermal cycle heating instruments, involve complex operations, and have long reaction times—factors that limit their use in the field (21–23). Therefore, it is necessary to develop a simple, rapid, sensitive, and specific diagnostic method for FMDV to enable timely detection and effective prevention of the disease.

In recent years, a variety of pathogen detection methods based on nucleic acid isothermal amplification technology have been developed to overcome the limitations of instruments and to shorten reaction times (24, 25). Loop-mediated isothermal amplification (LAMP) can achieve massive amplification of nucleic acid within 1 h at 65°C (26). However, its primer set design is extremely complex, and it is easy to produce false positive results due to aerosol contamination (27). As an isothermal nucleic acid amplification technology established in recent years, recombinase polymerase amplification (RPA) can complete the amplification at 37–42°C within 30 min (28, 29). Compared to LAMP, RPA is simpler and faster. RAA is a new isothermal amplification technology independently developed and modified by our country based on RPA, and the amplification reaction can be completed at a reaction condition closer to room temperature within 15–30 min. In recent years, RAA technology has been rapidly adopted in the diagnosis of various pathogenic microorganisms due to its advantages of simple primer design, rapid amplification, and high sensitivity (11, 30, 31). Nucleic acid test strip technology also offers benefits such as simple operation, result visualization, and rapid detection. The results can be determined using the strip within 10 min, making it more advantageous than conventional methods in terms of operation and equipment requirements.

Based on RAA and nucleic acid test strip technology, the FMDV nucleic acid RAA test strip method specific to the FMDV 3D gene was established in this study. The FMDV 3D gene is highly conserved across all seven FMDV serotypes. While experimental validation in this study focused on FMDV serotype O due to its epidemiological dominance in China, the strong sequence conservation of the 3D region strongly suggests that the assay has the potential for pan-serotype detection. The amplification of the target nucleic acid using the FMDV nucleic acid RAA test strip method can be completed within 30 min at 32°C, and the detection results can be observed within 5 min. The detection limits of plasmid and virus titers using the FMDV nucleic acid RAA test strip method were 10 copies/μL and 1 × 10^0^ TCID_50_/mL, respectively. Repeatability testing with the 10 copies/μL of standard plasmid across three replicates yielded consistent positive results, confirming robust intra-assay precision. In addition, 17 positive samples and 21 negative samples were tested to verify the practicability of the method. The results showed that the positive detection rate of this method was 100%, which was consistent with qPCR results. In summary, the FMDV nucleic acid RAA test strip developed in this study does not require precision instruments, enables rapid visual detection of nucleic acids, and is feasible for clinical field applications, especially in remote or resource-limited settings. However, a comprehensive investigation of reagent storage stability and operational tolerance under field conditions will be necessary for real-world deployment. These aspects require systematic evaluation in future validation studies. Furthermore, the practicality of the FMDV nucleic acid RAA test strip needs to be verified using more on-site clinical applications.

## Data Availability

The original contributions presented in the study are included in the article/supplementary material, further inquiries can be directed to the corresponding authors.
